# Supplementary material for: The diversity of cellular systems involved in carbonate precipitation by Escherichia coli
Source: PLoS One. 2025 Dec 16;20(12):e0319530. doi: 10.1371/journal.pone.0319530 (PMC12707652; doi:10.1371/journal.pone.0319530)
Supplement: S2 Fig — Only three ChaA transformants were obtained from the the pRPO::chaA expression construct transformed into E. coli. Prior sequence analysis has suggested that E85 (green) is involved in proton exchange [1] and the central acidic region between E199 – D208 (blue) is a Ca2+ binding domain [2]. While Ivey et al. (1993), suggested the protein contained 11 transmembrane a-helices [2], all but one (L17 – P37) overlap with the 10 domains identified by a Phyre2 structural analysis (grey) [3]. Sequencing of the chaA gene inserts revealed the following mutations: L221V, A236E, and S244P, all of which were associated with the sixth transmembrane domain. (PDF) [file pone.0319530.s003.pdf]

Supplemental Figure S2: Only three ChaA transformants were obtained from the the pRPO::*cha*A expression construct transformed into *E. coli*. Prior sequence analysis has suggested that E85 (green) is involved in proton exchange [1] and the central acidic region between E199 – D208 (blue) is a Ca^{2+} binding domain [2]. While Ivey et al. (1993), suggested the protein contained 11 transmembrane α-helices [2], all but one (L17 – P37) overlap with the 10 domains identified by a Phyre2 structural analysis (grey) [3]. Sequencing of the *chaA* gene inserts revealed the following mutations: L221V, A236E, and S244P, all of which were associated with the sixth transmembrane domain.

```
Klebsiella        mshaheavktrhkesslvfp vlalavlffwgssqslpvvi ainilalvgilssafsvvrh   60
Kluyvera          mthaqeavktrhketslifp lvalailffwgssqslpvvi ainamalvgilssafsvvrh   60
Shigella          msnaqeavktrhketslifp vlalvvlflwgssqtlpvvi ainllaligilssafsvvrh   60
Enterobacter      mthaqeavktrhketslifp vlalavlffwgsnqslpvvi ginilaligilssafsvvrh   60
Salmonella        mthaceavktrhketslifp vlalvvlflwgssqslpvvi ginilaligilssafsvvrh   60
WT                msnaqeavktrhketslifp vlalvvlflwgssqtlpvvi ainllaligilssafsvvrh   60
ChaA Mutatants    msnaqeavktrhketslifp vlalvvlflwgssqtlpvvi ainllaligilssafsvvrh   60
                  *::* *********:**:** ::**.:**:***.*:***** .** :**:************

Klebsiella        advlahrlgepygslilsls vvilevslisalmatgdaap tlmrdtlysiimivtgglvg   120
Kluyvera          advlahrlgepygslilsls vvilevslisalmatgdaap tlmrdtlysiimivtgglvg   120
Shigella          advlahrlgepygslilsls vvilevslisalmatgdaap tlmrdtlysiimivtgglvg   120
Enterobacter      advlahrlgepygslilsls vvilevslisalmatgdaap tlmrdtlysiimivtgglvg   120
Salmonella        advlahrlgepygslilsls vvilevslisalmatgdaap tlmrdtlysiimivtgglvg   120
WT                advlahrlgepygslilsls vvilevslisalmatgdaap tlmrdtlysiimivtgglvg   120
ChaA Mutants      advlahrlgepygslilsls vvilevslisalmatgdaap tlmrdtlysiimivtgglvg   120
                  ******************** ******************** ********************

Klebsiella        fslllggrkfatqymnlfgi kqylialfplaiivlvfpma lpganfstgqsllvavisaa   180
Kluyvera          fslllggrkfatqymnlfgi kqylialfplaiivlvfpts lpganftigqsllvalisaa   180
Shigella          fslllggrkfatqymnlfgi kqylialfplaiivlvfpma lpaanfstgqallvalisaa   180
Enterobacter      fslllggrkfatqymnlfgi kqylialfplaiivlvfpma lpganfstgqallvalisaa   180
Salmonella        fslllggrkfatqymnlfgi kqylialfplaiivlvfpma lpqanfstgqallvalisaa   180
WT                fslllggrkfatqymnlfgi kqylialfplaiivlvfpma lpaanfstgqallvalisaa   180
ChaA Mutants      fslllggrkfatqymnlfgi kqylialfplaiivlvfpma lpaanfstgqallvalisaa   180
                  ******************** *****************  : ** ***: **:****:****

Klebsiella        mygvflliqtkthqslfvye heddsddddphhgkpsahss gwhtawllvhlvaviavtkm   240
Kluyvera          mygvflliqtkthqslfvye heddsddddphhgkpsahss lwhavwlivhlvaviavtkm   240
Shigella          mygvflliqakthqslfvye heddsddddphhgkpsahss lwhaiwliihliaviavtkm   240
Enterobacter      mygvflliqtrthqnlfvye heddsddddphhgkpsahss lwhtvwlivhliaviavtkm   240
Salmonella        mygvflliqtkthqslfiye hedegdddnphhgkpsahss swhtvwllvhliavivvtkm   240
WT                mygvflliqtkthqslfvye heddsddddphhgkpsahss lwhaiwliihliaviavtkm   240
ChaA Mutants      mygvflliqtkthqslfvye heddsddddphhgkpsahss vwhaiwliihliavievtkm   240
                  *********::***.**:** ***:.***:**********  **: **::**:*** ****

Klebsiella        natpletllltsmnapvaftg flvallilspeglgalkavl nnqvqramnlffgsvlatis   300
Kluyvera          nanpletllltsmnapvaftg flvallilspeglgalkavl nnqvqramnlffgsvlatis   300
Shigella          nassletllldsmnapvaftg flvallilspeglgalkavl nnqvqramnlffgsvlatis   300
Enterobacter      naspletllssmnapvaftg flvallilspeglgalnavl nnqvqramnlffgsvlatis   300
Salmonella        naspletllltsmnapvaftg flvallilspeglgalkavl nnqvqramnlffgsvlatis   300
WT                nassletllldsmnapvaftg flvallilspeglgalkavl nnqvqramnlffgsvlatis   300
ChaA Mutants      naspletllldsmnapvaftg flvallilspeglgalkavl nnqvqramnlffgsvlatis   300
                  **. ***** ********** ****************:*** ********************

Klebsiella        ltvpvvtliafltgnelrfg lgapemvvmvaslvlchisf stgrtnvlngaahmalfaay   360
Kluyvera          ltvpvvtliafmtgnelmfg lgmpemivmvaslvlchisf stgrtnvlngaahlalfaay   360
```

```
Shigella          ltvpvvtliafmtgnelqfa lgapemvvmvaslvlchisf stgrtnvlngaahlalfaay   360
Enterobacter      ltvpvvtliafmtgndlsfa lgapemivmvsslvlchisf stgrtnvlngaahlalfaay   360
Salmonella        ltvpvvtliawvtgndlvfg lgapemivmvaslvlchisf stgrtnvlngaahlalfaay   360
WT                ltvpvvtliafmtgnelqfa lgapemvvmvaslvlchisf stgrtnvlngaahlalfaay   360
ChaA Mutants      ltvpvvtliafmtgnelqfa lgapemvvmvaslvlchisf stgrtnvlngaahlalfaay   360
                  **********::***:* *. ** ***:***:********* *************:******

Klebsiella        lmtifa      366
Kluyvera          lmtifa      366
Shigella          lmtifa      366
Enterobacter      lmtifa      366
Salmonella        lmtifa      366
WT                lmtifa      366
ChaA Mutants      lmtifa      366
                  ******
```


1. Fukaya F, Tanaka K, Waditee R, Tanaka Y, Nakamura T, Takabe T. (2010) Glultamate 85 is involved in the sodium/proton exchange activity of the *Escherichia coli* ChaA. *Bioscience, biotechnology, and biochemistry.* **74(5)**:1116-9.

2. Ivey DM, Guffanti AA, Zemsky J, Pinner E, Karpel R, Padan E, et al. (1993) Cloning and characterization of a putative Ca2+/H+ antiporter gene from *Escherichia coli* upon functional complementation of Na^+/H^+ antiporter-deficient strains by the overexpressed gene. *Journal of Biological Chemistry*. **268**:11296-303.

3. Kelley LA, Mezulis S, Yates CM, Wass MN, Sternberg MJE. (2015) The Phyre2 web portal for protein modeling, prediction and analysis. *Nature protocols*. **10(6)**:845-58.
