# Supplementary material for: The diversity of cellular systems involved in carbonate precipitation by Escherichia coli
Source: PLoS One. 2025 Dec 16;20(12):e0319530. doi: 10.1371/journal.pone.0319530 (PMC12707652; doi:10.1371/journal.pone.0319530)
Supplement: Attachment — Submitted filename: Reviewer Recommendation for PONE-D-25-05958R1.docx [file pone.0319530.s005.docx]

**Manuscript Number: PONE-D-25-05958R1**

**The diversity of cellular systems involved in carbonate precipitation by Escherichia coli**

**Recommendation: Accept as is**

Reviewer #1:

The reviewer reviewed the revisions made by the authors. The detailed review content are as follows:

1. The authors have made appropriate revisions and simplifications to the Discussion sections.

2. The authors have removed the phrase “data not shown” throughout the manuscript and added the data to supplemental materials.

3. The authors have updated or modified the captions of the corresponding tables as requested in the revised manuscript.

4. The authors have reviewed and modified the references throughout the manuscript.

The authors have made detailed revisions according to the reviewers’ comments.
The revised manuscript meets the requirements and is suitable for publication.
