# Supplementary material for: The diversity of cellular systems involved in carbonate precipitation by Escherichia coli
Source: PLoS One. 2025 Dec 16;20(12):e0319530. doi: 10.1371/journal.pone.0319530 (PMC12707652; doi:10.1371/journal.pone.0319530)
Supplement: Attachment — Submitted filename: Response to Reviewer Comments Final.docx [file pone.0319530.s006.docx]

Dear Dr. Basha,

Thank you for your feedback. We appreciated the constructive feedback from the reviewers and have addressed many of their questions below (our answers are in blue). Regarding the journal-specific requirments, we have:

1. Checked the manuscript for style requirements.
2. In the submission, under the Financial Disclosure section we answered “Yes” to the question “Did you receive funding for this work?”. Then, in the next section we proceeded to list the two funding sources. Is the issue because the “Yes” answer, and the list of funding sources, are in two different sections? If so, we apologize for the confusion. However, the listed funding sources are correct as written and no additional sources need to be added. This clarification has also been added to the new cover letter as requested.
3. The discussion of the funding has been removed from the acknowledgment section.
4. We have removed the phrase “data not shown” throughout the manuscript and added the data to supplemental materials. An extra section was added to Materials and methods describing how the protein gel image in the new supplemental figure was prepared.
5. A captionwas added for S1 Table at the end of manuscript.
6. We have reviewed the references. Only one change was made: Reference 51 was changed from a patent to the peer-reviewed paper that was the basis for the patent.

**RESPONSE TO REVIEWERS**

***Reviewer #1:*** I have reviewed the manuscript titled, “The diversity of cellular systems involved in carbonate precipitation by Escherichia coli”. The authors clearly lay out the importance of finding new ways to trap CO2 and present one possible route – MICP. They identified genes possibly involved in the E. coli MICP pathway, classified into four primary groups, and further investigated some of the identified genes to better understand their possible role.

We thank the reviewer for their positive comments on the manuscript.

While the bulk of the manuscript has sound conclusions, I still have some lingering questions, especially about the iron metabolism and efflux pump results and conclusions, therefore I would recommend this manuscript for minor revisions. Below are my points of concern.

- Some of the tables and figures need some further clarification in their captions. The Table 1 caption should include that the genes that are highlighted in gray and bolded are those that underwent further investigation. Additionally, it would be useful if Table 1 had another column which stated if the carbonate production was higher or lower in that strain.

We have updated the Table 1 caption as requested and to more clearly described its content, highlighted that the identified genes lack the CaCO3 precipitation phenotype.

- Figure 2C should indicate that the dash line is the pH at which SI is reached.

The caption of Figure 2 has been updated to include this change.

- Figure 4 should clarify what the different colors are in the two graphs and if only the WT strain was used in these studies.

This has been clarified in the figure legend.

- In the section on central metabolism, how were the representative genes selected for further study over the others? It would be useful to have a brief explanation for this.

This was clarified in the manuscript.

- The authors look at plating versus liquid culturing methods, but what calcium source is used in the liquid cultures shown in Fig. 2?

The figure legend was updated to emphasize calcium-propionate was the calcium substrate in liquid cultures.

- When focusing in on complex I, the authors conclude that the data in Fig. 3 and Fig. 2 are correlated, but this is not a true statement - there are conflicting results between Fig. 2A and Fig. 3. For example, in Fig. 3 for both acetate and propionate, the level of insoluble Ca2+ is very low and almost indistinguishable for all three strains tested. However, according to Fig. 2A, levels of the *nuoB* knockout were noticeably less than the other two strains. Please check the color-coding scale in Fig. 2A to ensure that the results between liquid and solid culturing do actually correlate.

Figure 2A was a qualitative comparison of calcification on solid media, whereas Figure 3 allowed quantification in liquid culture – this language is used (qualitative versus quantitative) in the legend for each figure. As the the liquid quantification assay was quantitative, it was a lot more sensitive and could detect the initiation of calcification earlier that the plate-based assay. We have clarified this in the manuscript.

- The iron metabolism section is underdeveloped in my opinion. The authors nicely tested the shift in timing of CaCO3 precipitation with the WT strain, but did not test any of the strains with the knockouts that they have found may be involved. Since three of the genes found are transporters, this would have helped to solidify a link between iron transport and CaCO3 precipitation. The authors should either perform this experiment or comment on why this was not tested.

We agree with the reviewer that using one of the iron knock-out strains would have provided supporting evidence for the proposed; however, these experiments are extremely challenging to set up (taking more than a week per run) and comparisons with the knock-outs were not considered at the time as the products turned brown, limiting their usefulness as fillers. At the current time we do not have the capability to carry out additional experiments with this system, but have captured the thought that such experiments could help disentangle this question in the discussion as follows:

*Ln 484: “Additional continuous culture experiments using some of the Keio strains associated Fe metabolism would allow the exploration of this relationship further.”*

- Based on what is shown in the efflux pumps section, I am not convinced that zntB is the likely Ca2+ exporter involved in the MICP pathway. The data shown in Fig. 6 for the control and yrbG/can/cynT does nicely show the validity of their expression approach, however it does not conclusively show that zntB is functioning as a Ca2+ transporter. The levels should not have already been that high for uninduced zntB. The explanation about the promoter being leaky is not a proper explanation for this observation considering the other plasmids incorporated the same promoter and this issue was not encountered. Further explanation needs to be included here. Additionally, zntB cannot be named as THE exporter when the other two efflux pumps identified weren’t even tested in the same fashion just because less was known about these two pumps. Please comment on the lack of testing of the other two pumps.

The decision to look at ZntB compared to the other transporters was discussed in the manucrscipt (Ln 346*: “Given the greater level of understanding of zntB function (compared to mdtH and mdtL [47]), we decided to study its effect on carbonatogenesis by cloning into an expression vector (pPRO24) in E. coli.”*

We have included the SDS-PAGE data within supplemental materials, demonstrating a slight increase in expression of *can* in the uninduced vector. As we stated in the manuscript, it is much easier to see the impact on the soluble fraction (which contains *can*), but is in support of the leaky hypothesis. The other reviewer suggested that the half-life of ZntB, as a membrane associated-protein would be higher, which could amplify this affect. To address the concerns of the reviewer, we have added additional information to state that the proposed leakiness is a hypothesis, rather than demonstrated effect. We have also clarified throughout the manuscript that ZntB was the only transporter tested, that it only hints at Ca2+ transport, and that the role of the other transporters needs to be more fully explored.

***Reviewer #2:*** Calcium stress-dependent Microbially Induced Carbonate Precipitation (MICP) in E. coli is a novel and promising approach for trapping atmospheric CO2 as calcium carbonate (CaCO₃). Unlike ureolysis-based MICP, this system avoids ammonia-related issues, offering a cleaner alternative for industrial applications; however, the yield of precipitated CaCO3 is presently quite low.

With the aim to optimize the process, the authors of the present study screened a E. coli knockout library and identified 54 genes whose inactivation affects CaCO3 precipitation. These genes are involved in central metabolism, iron metabolism, cell architecture, and ion transport systems.

**General comments**

The manuscript is generally well written and detailed, although several paragraphs in the Results and Discussion sections are a little “verbose”: overall, the MS may benefit from a more concise style.

We appreciate the feedback, and a review has identified several areas where the language could be streamlined or reiterated a previously mentioned point. We have attempted to tighten up the discussion section and hope the reviewer finds the changes appropriate.

Titles of the Results sub-sections are appropriate, but quite generic and scarcely informative.

We attempted to add more information, but this ended up creating more confusion and adding to the overall verbose nature of the document, so no changes we made.

The work is well done from a technical perspective: experiments are well conceived and generally well performed (with few exceptions: see below). Data reported generally support the Authors’ conclusions and speculations. Still, most issues are not examined in great detail.

We thank the reviewer and agree that much work needs to be done. To this regard, we believe that this manuscript is a jumping off point for us to study certain aspects of carbonatogenesis in more detail. We hope that the extensive list of genes that were identified may help others identify connectinos to their own work within the MICP field.

The graphic quality of the figures is generally adequate, with complete and descriptive legends (with minor exceptions: see below).

In the text there are way too many references to data not shown, which in my opinion should be included as Supplementary Materials.

We have either removed or provided this data in the supplemental materials section.

Methods are well detailed.

The relevant Literature is cited in the appropriate context.

The novelty degree of the paper is not overwhelming, but is nonetheless significant, since it identifies several new potential genetical targets/cellular processes which may be manipulated to enhance CO2 fixation by MICP in E. coli: namely, i) CO2 uptake; ii) Ca2+ efflux; iii) ammonium generation by amino acids metabolism; iv) enrichment of MICP-promoting polymers around the cell surface.

We thank the reviewer, and hope the paper and our approaches in *E. coli* will help stimulate new directions within the field.

Minor Remarks

• The KO E. coli library was screened by a rapid qualitative plate visual assay to evaluate any genetic impacts on carbonatogenesis, allowing the identification of 54 mutants with altered CaCo3 precipitation phenotype. However, only the list of these mutants is reported in Table 1, with no results clarifying to which extent carbonatogenesis was deficient (or possibly enhanced) in these strains.

Yes, this was an oversight. We have clarified within the table and manuscript that we targeted our analyses to mutants that lacked the carbonategenesis phenotype.

Several representative mutants (indicated in bold in Table 1) were selected for further characterizations, including (for some but not all the strains) a more quantitative assay in liquid medium to precisely evaluate the amount of precipitated carbonate (Fig. 3 and Fig. 5). It would be useful if the quantitative assays were performed for all the selected mutants.

The AtpF and SdhC mutants did not produce carbonate under any of the conditions tested, and additional liquid assays would not have generated any comparative data. We have clarified this thought process in the paper.

• In fig 2B-C, the authors claim that the onset of carbonate precipitation in liquid medium occurs when a notable bump in OD600 and a simultaneous drop in the pH of the culture are registered. However, these bumps/drops are quite modest for most strains (including the wild type, dark blue symbols), at least in this particular experiment, whereas they are much more evident in fig. 4.

The experiment in Figure 4 was continuous culture, where data loggers allow data to be recorded at 1 minute intervals. This allows a much finer resolution of the ‘bumps’ that indicate the onset of carbonatogenesis. The data in Figure 2 were obtained individually, either in side-arm flasks or collecting samples for pH measurements. We have clarified these differences to better explain this discrepancy.

Role of Iron metabolism.

• The authors list several mutants in iron metabolism with reduced CaCO3 precipitation, but they report no qualitative or quantitative analysis about the amount of this reduction. These mutant were no further characterized.

• The role of iron on the timings of carbonatogenesis in liquid medium was evaluated by continuously measuring the pH medium (with the onset of CaCO3 crystal accumulation coinciding with a sudden drop in pH). The experiment was performed at 24°C (lower temperatures reduce growth rate and improve temporal resolution of the assay). In other experiments (e.g. fig. 2) cell are grown at 37°C. Has temperature any impact at all on the efficiency of CaCO3 precipitation?

As the continuous culture system we use only operates at room temperature, so we do not have any data on the impact of growth at 37°C under similar conditions. Nonetheless, prior work has not indicated a significant change in overall CaCO_3_ yield (for example, see Breley *et al*., 2023, doi/full/10.1080/ 01490451.2022.2115169) between different growth temperatures.

• It is unclear what the series plotted in the upper (untreated) and lower (+FeCl3) panels of Fig4 represents: various experimental replicates for wild type strains? Different strains? Nothing is specified, either in the legend or in the main text.

This oversight has been corrected.

• Addition of FeCl3 to growth medium clearly anticipates the timings of carbonatogenesis. Does it also affect the amount of precipitated CaCO3?

Visual inspection of the total precipitate did not indicate any change, other than the color change as described. As we did not collect samples to make quantitative comparisons at the time, we are unable to carry out such quantitation. As the time and effort needed to establish independent continuous cultures, each lasting over 4 days, this would be a significant burden to repeat. Nonetheless, it is an interesting question and one that we have added to the discussion.

• Can FeCl3 supplement improve carbonatogenesis in mutants deficient in iron metabolism?

We used calcium propionate in Figure 2, and calcium succinate in Figure 4, which is why this observation was much more pronounced. It is also important to clarify that the data in Figure 4 was obtained from continuous culture, which allows us to record data every minute, as opposed to the growth curves in Figure 2, which are taken hourly for the first 8 hours. This means that the discrete bump is only observable when much more detailed data are recorded. This has been clarified in the manuscript.

The reviewer is correct; however, Figure 4, which looks at the impact of iron metabolism only occurred in the WT strain. As none of the iron metabolism mutants produced carbonates, it means that no data would have been obtained for those mutants in this assay. It is therefore unclear what information or new perspectives could be gained by examining these deficient mutants. This has been clarified in the manuscript.

Our continuous culture experiments occur at room temperature, as our bioreactor does not allow us to modify the temperature to 37°C. As such, it is not possible to compare the two temperature ranges and outside the scope of this project. Nonetheless, we have modified the language in the manuscript to highlight this discrepancy. Future analyses could examine this temperature impact, and we have included that in the discussion.

Efflux pump

• Consistently with the key role of calcium export in carbonatogenesis, the authors identified in their preliminary screen several ion efflux pumps which may drive the process, in particular Zntb, which was previously involved in zinc homeostasis (whereas no Ca2+ binding has been reported so far in literature). Unfortunately, no quantitative data for carbonatogenesis relative to the ΔzntB null mutant are shown in the manuscript.

ZntB was identified in our original screening assay; however, our initial quantification assays were focused more on the role of cellular metabolism in carbonatogenesis. As ZntB was the only cation transporter identified in our screen, we decided to examine this via over-expression compared to previously identified transporters that had been shown to alter the calcification phenotype. We have clarified this logic flow in the manuscript.

• Instead, the authors convincingly show how the overexpression of zntB from a multicopy plasmid under an inducible promoter significantly increased (3-fold) CaCo3 precipitation, whereas no effect is observed upon overexpression of yrbG (a known calcium transporter not involved in carbonatogenesis: Fig. 5).

However, no differences were registered for zntB between the induced and uninduced conditions, which may result from the leaky promoter coupled with a multicopy plasmid, as suggested by the author. Nonetheless, the dosing effect of induction on CaCO3 precipitation is clearly detectable when carbonic anhydrases are overexpressed using the same combination of plasmid and promoter: in this case, the difference may be due to the longer half-life of ZntB (a membrane protein) relative to the shorter half-life of the cytoplasmic carbonic anhydrases. Since SDS-PAGE expression data are cited but not shown, it is unclear which levels of overexpression are actually achieved for Zntb under uninduced/induced conditions.

This is an interesting idea that we did not consider. We have included the SDS-PAGE data to demonstrate the increase in expression of ZntB and yrbG, and have mentioned the possibility of the increased half-life within the manuscript.

• Did the authors test a low copy plasmid or another inducible promoter for their expression system?

No other plasmids were tested; however, this is an interesting idea and we will look into it as part of our continuing exploration of this gene system.
